# Is Interleukin 17 (IL-17) Expression A Common Point in the Pathogenesis of Depression and Obesity?

**DOI:** 10.3390/jcm9124018

**Published:** 2020-12-12

**Authors:** Katarzyna Bliźniewska-Kowalska, Bernadeta Szewczyk, Małgorzata Gałecka, Kuan-Pin Su, Michael Maes, Janusz Szemraj, Piotr Gałecki

**Affiliations:** 1 Department of Adult Psychiatry, Medical University of Lodz, 91-229 Lodz, Poland; piotr.galecki@umed.lodz.pl; 2 Department of Neurobiology, Maj Institute of Pharmacology Polish Academy of Sciences, 31-343 Cracow, Poland; szewczyk@if-pan.krakow.pl; 3 Department of Psychotherapy, Medical University of Lodz, 91-229 Lodz, Poland; malgorzata.galecka@umed.lodz.pl; 4 An-Nan Hospital, China Medical University, Tainan 709, Taiwan; cobolsu@gmail.com; 5 Department of Psychiatry, Faculty of Medicine, Chulalongkorn University, Bangkok 10330, Thailand; dr.michaelmaes@hotmail.com; 6 Department of Medical Biochemistry, Medical University of Lodz, 92-215 Lodz, Poland; janusz.szemraj@umed.lodz.pl

**Keywords:** depression, obesity, IL-17, neuro-immune, inflammation

## Abstract

(1) Background: Activated immune-inflammatory pathways play an important role in the pathogenesis of depression and pathological obesity. Obesity might promote production of cytokine interleukin 17, which plays a significant role in neuro-immune reactions. The study aimed at assessing the relationship between Body Mass Index (BMI) and IL-17 expression, taking into account the clinical psychiatric variables in patients with depression. (2) Methods: A total of 125 participants took part in the study (95 depressed patients, 30 healthy controls). Data concerning the course of depressive disorders and BMI were collected. The severity of depressive symptoms was assessed using the Hamilton Depression Rating Scale (HDRS). Reverse transcription polymerase chain reaction (RT-PCR) was used to assess IL-17 gene expression at the mRNA levels, while enzyme-linked immunosorbent assay (ELISA) was used to assess IL-17 expression at the protein level. (3) Results: Patients with more hospitalizations showed significantly higher IL-17 mRNA expression levels and higher BMI. However, no correlation between BMI and IL-17 expression was found in depressed patients. (4) Conclusions: Our study revealed that BMI does not affect IL-17 expression in patients with depression. However, further studies should be conducted to evaluate the effects of IL-17 inhibition on adipose tissue and vice versa.

## 1. Introduction

Patients suffering from mood disorders often experience significant weight gain or weight loss, due to their medical condition, lifestyle changes and pharmacological treatment. Body weight is associated with the body’s long-term energy balance, which is related to the ratio of energy intake to energy expenditure. Both the amount of absorbed energy and the level of metabolism, which are the main determinants of energy expenditure, are under control of the brain. The activity of subcortical centers is mainly related to the instinctive search for food and the stimulation of the reward system following food consumption. An important role in the energy balance is also played by the cerebral cortex, which is responsible for psychological components of food consumption regulation, e.g., social eating [1]. No wonder then that psychotropic medication affects appetite. Weight gain can be a side effect of commonly used antidepressants such as mirtazapine, mianserine or paroxetine. This effect might be exacerbated by the addition of mood stabilizer or antipsychotic medication to augment the therapy of depression [2].

However, not only pharmacotherapy, but also depressive disorders themselves might lead to overweight or obesity. Some studies suggest that symptoms of depression, including shortened sleep, sedentary behavior and depressed mood may overlap as predictors of obesity [3]. Not all cases of depression are associated with decreased appetite. According to Angst et al. 2002, in about 5% of clinical cases atypical depression was diagnosed [4]. Atypical depression is characterized by vegetative symptoms, female predominance, early onset and trait-like interpersonal sensitivity [5]. Primary dimensions of vegetative symptoms in patients with atypical depression are weight gain, increased appetite and hypersomnia. Studies have shown that “atypically” depressed patients have significantly higher rates of binge eating comorbidity and they are more impulsive than those in the non-atypical depression group [6].

Both depression and obesity are diseases of civilization, which constitute very serious social problems. Their high prevalence- about 350 million depressed patients and 500 million obese subjects [7,8] and enormous impact on everyday life result in significant economic implications, reduction of participation in labor force and absenteeism [9,10].

Depression and obesity are disorders of stress, which regardless of its source, is associated with cascade of biological changes in the body [11]. Dysregulation of hypothalamic-pituitary-adrenal (HPA) axis, also called the stress axis, is associated with activated immune-inflammatory pathways [12,13].

**Figure 1:**
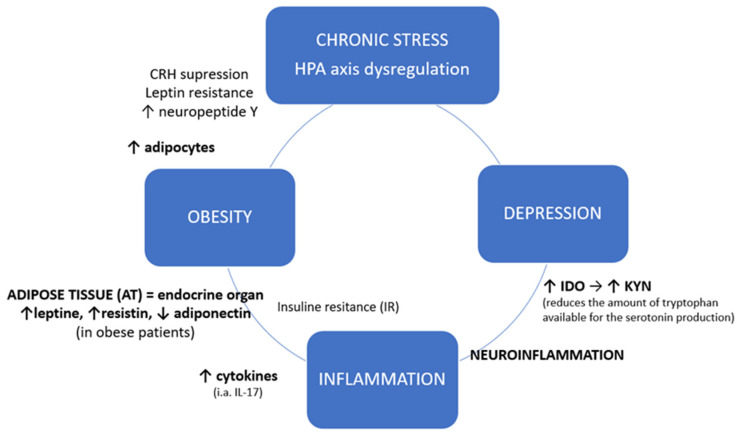
Chronic stress and inflammatory crosslink between obesity and depression. HPA axis: hypothalamic-pituitary-adrenal axis; CRH: Corticotropin-releasing hormone, corticoliberin; IDO: Indoleamine-pyrrole 2,3-dioxygenase; KYN: Kynurenine.

Chronic stress and coexisting inflammation might serve as a common point between obesity and depression. Hyperactivation of HPA axis leads to corticoliberin suppression, leptin resistance and increased neuropeptide Y (NPY) release, thus to increased appetite [14]. Neuropeptide Y in turn promotes the growth and differentiation of adipocytes [15]. Adipose tissue (AT) is an endocrine organ that produces and releases variety of proteins and therefore serves as a connection between peripheral organs and central nervous system (CNS) [16]. Proteins specifically secreted from AT are called adipokines, the most important representatives of which are leptin, resistin and adiponectin [17]. Leptin and resistin work in a pro-inflammatory way, where aponectin, which is reduced in obese patients, plays predominantly an anti-inflammatory role. Especially resistin increases the secretion of cytokines [18]. Increased secretion of TNF-alfa, IL-6 and IL-1beta locally by macrophages in infiltrated adipose tissue causes insulin-resistance (IR), which in short-term is beneficial in defending against pathogens, by a decrease in nutrient storage and increase of glucose concentration [19]. Permanent inflammation, leading to chronic IR state, results in metabolic syndrome and serious health problems, like type 2 diabetes [20]. Obesity can be considered as a low-grade chronic inflammation state, in which adipose tissue (AT) becomes heavily infiltrated by immune cells, i.e., T lymphocytes. T cell distribution varies in obese adipose tissue, with a tendency toward higher CD8+ to CD4+ ratios, especially in visceral adipose tissue [21]. However, adipose tissue also expresses high levels of circulating interleukin 17 (IL-17A), which is mainly secreted by T helper 17 (Th17, CD4+) cells [22,23,24] (Figure 1).

As shown in Figure 1, besides their local action, systemic cytokines derived from adipose tissue may also enter the central nervous system which leads to neuroinflammation [25]. Inflammatory factors cause excessive activation of IDO (indoleamine-2,3-dioxygenase), an enzyme present in microglia, astrocytes and neurons, catabolizing the conversion of tryptophan into the neurotoxic kynurenine (KYN), thus reducing the availability of tryptophan for the production of serotonin. Kynurenine, in turn, influences the intensification of neurodegenerative processes [26,27]. Altered neurogenesis and neuroplasticity and serotonin deficit contribute to depression [28].

Scientific data suggest that IL-17 and T helper-17 (Th17) phenotype may also play an important role in neuroimmune interactions in depressed patients [29]. IL-17 expression may serve as a link between the etiopathogenesis of depressive disorder and pathology of obesity. A question can be raised whether IL-17 expression can be affected by weight change in depressed individuals.

The aim of the study was to assess the relation between the Body Mass Index (BMI), an indicator of overweight and obesity, and the level of IL-17 expression, taking into account also psychiatric clinical variables.

## 2. Experimental Section

### 2.1. Participants and Data Collection

A total of 125 participants took part in the study. The study group included 95 patients (68 F, 27 M) with the diagnosis of a depressive episode or recurrent depression disorder (rDD) (F32 and F33, respectively, according to ICD-10 criteria) [30]. The control group consisted of 30 healthy volunteers (23F, 7M) with a negative history for mental disorders. Table 1 shows demographic and clinical characteristics of both studied groups. The exclusion criteria were as follows: other psychiatric diagnoses than depressive disorders, serious neurological or somatic diseases (including autoimmune diseases) that could affect the expression of IL-17, abuse and addiction to psychoactive substances.

Individuals taking part in the study were native Poles from central Poland (not related). They were chosen for the study group at random without replacement sampling. Participation in the study was voluntary. Written informed consent was obtained from each subject according to the study protocol that had been approved by the Bioethical Committee of the Medical University of Lodz (No. RNN/833/11/KB).

**Figure 2:**
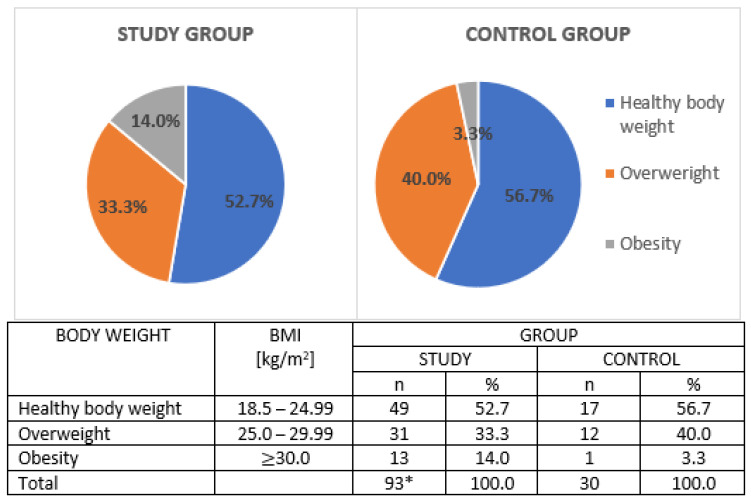
Body weight categories in studied groups; BMI: Body Mass Index; *n*: sample size; %: percentage. *: 2 patients from study group with BMI under 18.5 were excluded from the analysis.

Survey data, i.e., age, gender, calculated BMI (Body Mass Index) were obtained from all study participants. Qualification to a specific weight category, i.e., obesity, overweight, healthy body weight etc. was based on World Health Organization (WHO) BMI criteria [31] (Figure 2). There was no statistically significant difference between the study and the control group depending on body weight category (χ2 = 2.614; *p* = 0.271).

**Figure 3:**
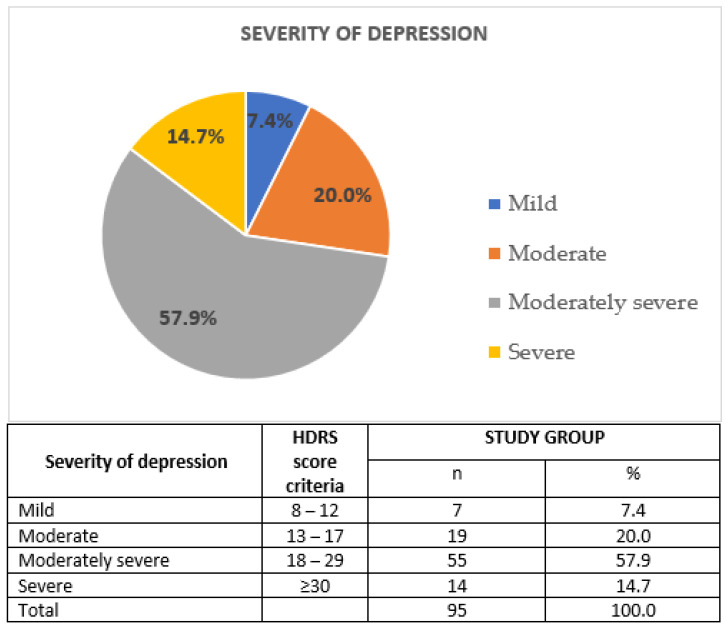
Severity of depression in the study group; HDRS: Hamilton Depression Rating Scale; *n*: sample size; %: percentage.

The mental state of patients from the study group was assessed on the day of inclusion by a qualified psychiatrist. Participation in the study was not associated with any change in the antidepressant therapy. Data concerning the course of depressive disorder were collected by means of Composite International Diagnostic Interview (CIDI) [32], also taking into account the duration of the disease (in years), number of depressive episodes and number of psychiatric hospitalizations. Moreover, the severity of depressive symptoms was assessed using Polish adaptation of 17-item Hamilton Depression Rating Scale [33]. Cronbach’s alpha (tau-equivalent reliability) for this scale was 0.70; the sensitivity coefficient was 0.78 and the test relevance coefficient was 0.75 [33,34]. Figure 3 illustrates the severity of depression in the study group. Hospitalized patients were enrolled to our study; hence, majority of them had moderately severe or severe depressive episodes.

Peripheral venous blood samples were collected from all the participants. RT-PCR was used to assess interleukin 17 (IL-17) gene expression at the mRNA level, while ELISA was used to assess interleukin 17 (IL-17) expression at the protein level. The obtained results were subjected to statistical analysis in order to determine the correlation between gene expression and clinical and sociodemographic data.

### 2.2. Interleukin 17–Protein Expression

#### 2.2.1. Determination of Protein Concentration

Serum total protein concentration and analytical curve for serum albumin were determined. Both the examined samples and the reference samples ran parallelly in three repetitions. Sample absorbance was measured using Multiskan Ascent Microplate Photometer (Thermo Labsystems, Philadelphia, PA, USA) at λ = 562 nm and total protein concentration was calculated from the standard curve equation.

#### 2.2.2. Enzyme-Linked Immunosorbent Assay (ELISA)

The concentration of IL-17 protein was determined in the serum using Human IL-17 Quantikine ELISA Kit according to the protocols provided by the manufacturer. β-actin was used for endogenous control of protein concentration in the samples and determined with the help of Human Actin Beta (ACTb) ELISA Kit (BMASSAY) based on the manufacturer’s recommendations. The absorbance of the samples was measured using Multiskan Ascent Microplate Photometer (Thermo Labsystems, Philadelphia, PA, USA) at λ = 450 nm. Analytical curves were made for the analyzed proteins to determine protein concentration.

### 2.3. Interleukin 17 Gene–mRNA Expression

#### 2.3.1. Total RNA Isolation

Total RNA isolation from the patients’ peripheral blood lymphocytes was performed using InviTrap Spin Universal RNA Kit (Stratec molecular, Berlin, Germany) based on the manufacturer’s recommendations. Absorbance was measured using a spectrophotometer (Picodrop) at λ = 260 nm in order to determine total RNA concentration. Isolated RNA was stored at 70 °C.

#### 2.3.2. Quality Analysis of Isolated RNA

The quality of total RNA was checked with Agilent RNA 6000 Nano Kit (Agilent Technologies) in accordance with the manufacturer’s recommendations. A total of 1 µL of RNA 6000 Nano dye was added to a test tube containing 65 µL of Agilent RNA 6000 Nano gel matrix and then centrifuged (10 min, 13,000× *g*). The gel-fluorescent dye mixture was applied on the surface of Nano chip placed in a workstation. Then, 5 µL of RNA Nano marker was added to selected pits. Isolated samples of RNA and RNA size marker were subjected to denaturation (2 min, 70 °C), and then 1 µL of the sample was pipetted to selected pits of the Nano chip and mixed (1 min, 2400 rpm). The quality of isolated RNA was checked using 2100 Bioanalyzer (Agilent Technologies). The level of degradation of total RNA was determined with the use of an electrophoretogram and RNA Integrity Number (RIN) values were recorded. Only the samples with RIN value > 7 were subject to further analysis.

#### 2.3.3. RT-PCR Reverse Transcription

RT reaction was carried out using TaqMan^®^ RNA Reverse Transcription Kit (Applied Biosystems) based on the manufacturer’s recommendations, using specific Hs00174383_m1, Hs04194366_g1 probes, respectively, for IL 17 and RPL13A genes, delivered by Applied Biosystems. The samples were incubated (30 min, 16 °C and 30 min, 42 °C) in a thermocycler (Biometra). Reverse transcriptase was inactivated (5 min, 85 °C) and the obtained cDNA was stored at 20 °C.

#### 2.3.4. Real-Time PCR Reaction

Real-Time PCR reaction was conducted using TaqMan^®^ Universal PCR Master Mix, No UNG (Applied Biosystems) according to the protocol provided by the manufacturer. The Ct comparative method was used to calculate the relative expression of mRNA of the studied genes [35]. The level of IL-17 gene expression in particular tissues was normalized in relation to RPL13A reference gene.

Each target probe was amplified in a separate 96-well plate. All samples were incubated at 50 °C for 2 min and at 95 °C for 10 min and then cycled at 95 °C for 30 s, at 60 °C for 30 s and at 72 °C for 1 min; 40 cycles were performed in total. Fluorescence emission data were captured and mRNA levels were quantified using the critical threshold (Ct) value. Analyses were performed with ABI Prism 7900 HT (SDS Software). No RT and no template cDNA controls were performed with each assay. Relative gene expression levels were obtained using the ∆∆Ct standard 2-∆∆ct calculations and expressed as a fold change of the control sample [36].

### 2.4. Statistical Analysis

STATISTICA 12.0 PL software was used to perform a statistical analysis of the results. The statistical analysis was performed with the use of both descriptive and inferential statistics. The qualitative characteristics of the groups of affected patients and healthy controls were expressed as frequencies and shown as percentages. An arithmetical mean ($\bar{x}$) was calculated in order to characterize the average values of quantitative features. Statistical dispersion measures included the values between the minimum and the maximum and standard deviation (SD). The consistency of the distributions of the analyzed quantitative variables with normal distribution was tested using the Shapiro–Wilk test. The distributions differed significantly from the normal distribution, so non-parametric tests were used to compare the means. The Mann–Whitney test for independent samples was used to compare the means of individual variables in the study group and in the control group. Critical value for Z score for *p* < 0.05 is 1.96 (Z_0.05_ = 1.96). Spearman’s rank correlation coefficient was used to assess the relationship between quantitative variables and its significance was assessed by Student’s *t*-test. The chi-square test (χ2) of independence was performed to compare the incidence among men and women in the study and control groups.

The differences between the means and the relationships between the variables for which the calculated test value was equal to or greater than the critical value read from the appropriate tables, with the appropriate number of degrees of freedom and error probability *p* < 0.05, were considered statistically significant.

## 3. Results

### 3.1. Body Mass Index (BMI)

The compared groups did not differ statistically (*p* > 0.05) regarding gender or BMI. Women dominated in both, the study and the control group. Fourteen percent of depressed patients from the study group were obese (BMI ≥ 30 kg/m^2^) and almost 1/3 of them (33.3%) overweight (BMI 25.0–29.9 kg/m^2^). Among healthy volunteers 40% were overweight and 3.3% obese. The characteristics of the studied groups is presented in Table 1.

Comparison of BMI in men and women from the study group showed a statistically significant difference (*p* < 0.001). The average BMI in men turned out to be significantly higher than in women: 26.9 ± 3.40 vs. 24.3 ± 5.21. No such relationship was found in the control group.

A statistically significant positive relationship between BMI and age was found in patients with depression (*p* < 0.05). However, the relationship was weak because the Spearman’s rank correlation coefficient was 0.210. The relationship between the BMI and the number of hospitalizations was also statistically significant (ρ = 0.237, *p* = 0.021). Patients with a higher BMI were significantly more often hospitalized as compared with patients with a lower BMI. The size of the BMI did not correlate statistically with other clinical variables (*p* > 0.05). In the healthy (control) group, there was also a statistically significant positive correlation between BMI and age (ρ = 0.438, *p* = 0.020). Although it was not statistically significant, a moderately severe depressive episode was observed much more often in obese (69.2%) patients than in patients with normal (healthy) body weight (59.2%) and overweight (48.4%).

### 3.2. Interleukin 17 Expression

Statistically significantly higher IL-17 expression was found at both protein and mRNA levels in depressed patients (Table 2).

Comparison of IL-17 protein and mRNA expression level in men and women from the study group and from the control group did not show a statistically significant difference (*p* > 0.05).

**Figure 4:**
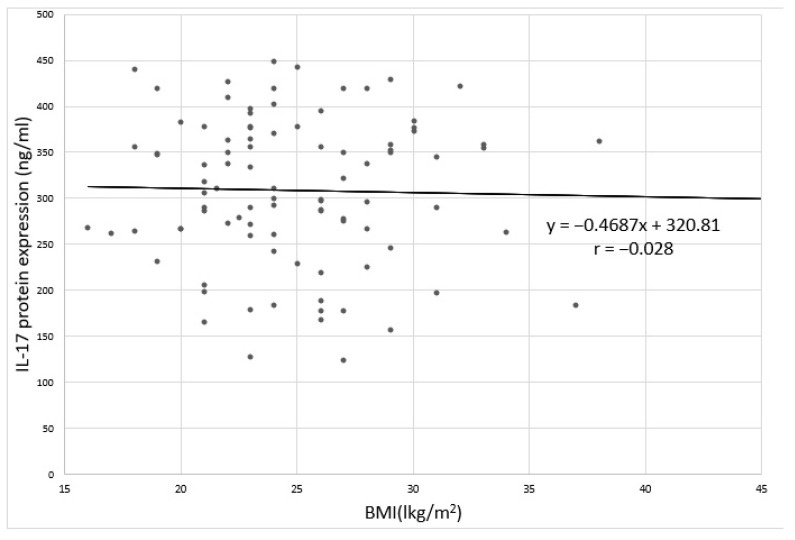
Correlation between IL-17 protein expression and BMI in depressed patients;. r: Pearson’s linear correlation coefficient.

**Figure 5:**
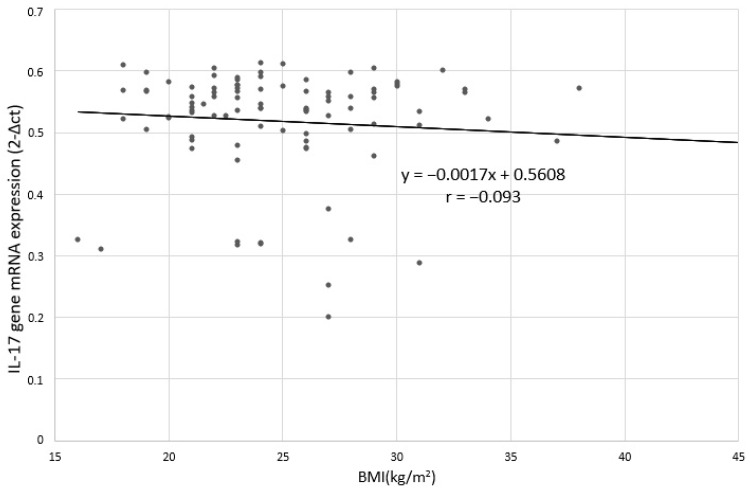
Correlation between IL-17 gene mRNA expression and BMI in depressed patients; r: Pearson’s linear correlation coefficient.

No statistically significant relation was found between the level of IL-17 expression and BMI in depressed patients (Figure 4 and Figure 5). Spearman’s rank correlation coefficient for the correlation of IL-17 protein expression and BMI in depressed patients was-ρ = −0.062; *p* = 0.744 and for the correlation between IL-17 mRNA expression and BMI in depressed patients was - ρ = −0.039; *p* = 0.382.

**Figure 6:**
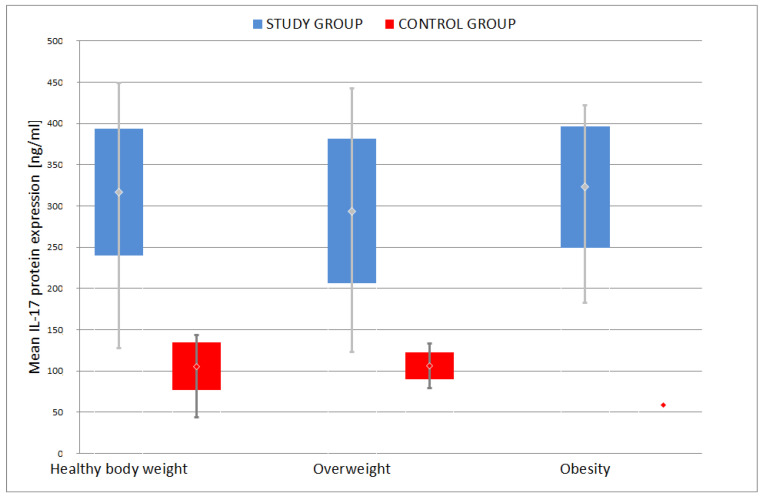
Comparison of mean IL-17 protein expression among different weight groups.

Further analysis of data in depressed patients indicated that there was no statistically significant difference between the mean IL-17 protein expression depending on their body weight: healthy body weight, overweight and obese (*p* = 0.182). However, it is worth noting that the highest average was recorded in obese patients: 304.1 ± 99.8, much lower in patients with healthy body weight: 262.3 ± 115.0, and the lowest in the group of overweight patients: 241. 5 ± 113.2 (Figure 6).

**Figure 7:**
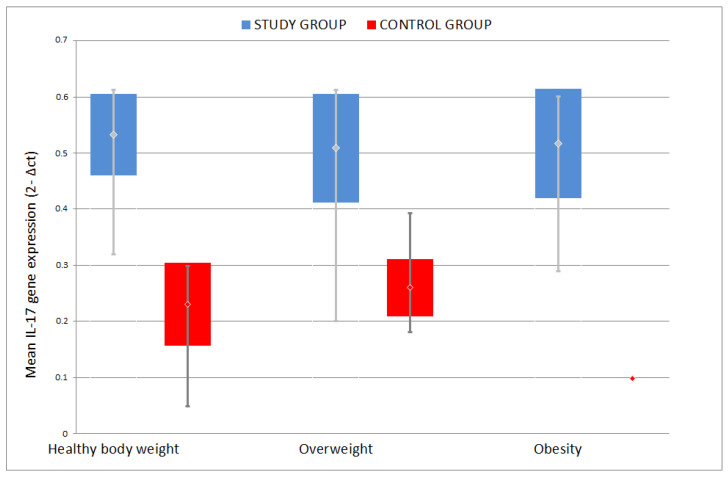
Comparison of mean IL-17 gene mRNA expression among different weight groups.

No statistically significant difference was found between the mean IL-17 gene mRNA expression among the groups categorized according to body weight (*p* = 0.242). Similarly, the highest mean was recorded in obese patients: 0.487 ± 0.146, lower in patients with normal body weight: 0.455 ± 0.152, and the lowest in the group of overweight patients: 0.439 ± 0.142 (Figure 7).

**Figure 8:**
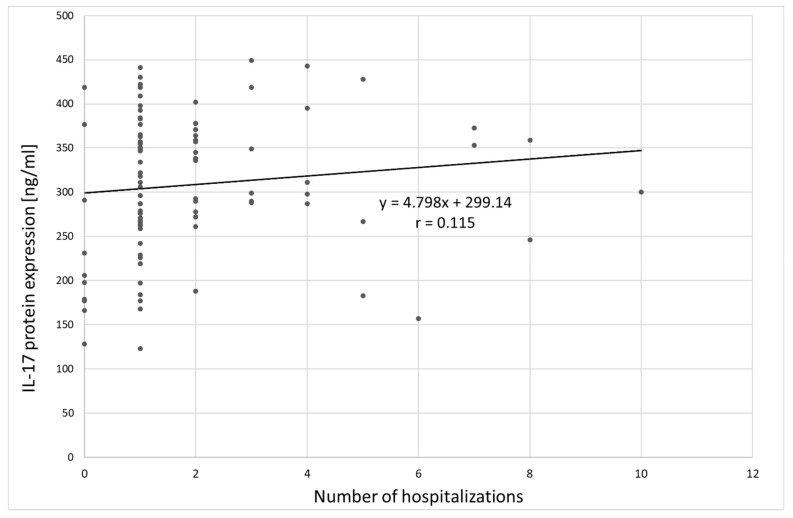
Correlation between IL-17 protein expression and number of hospitalizations;. r: Pearson’s linear correlation coefficient.

Analysis of the relation between IL-17 protein expression and clinical variables in the group of patients with depression showed only a statistically significant correlation between IL-17 protein expression and the number of hospitalizations (Spearman’s rank correlation coefficient ρ = 0.209; *p* = 0.042), indicating that patients with multiple hospitalizations had higher IL-17 protein expression (Figure 8). The correlation between IL-17 protein expression and age was not statistically significant (ρ = 0.144, *p* = 0.163).

**Figure 9:**
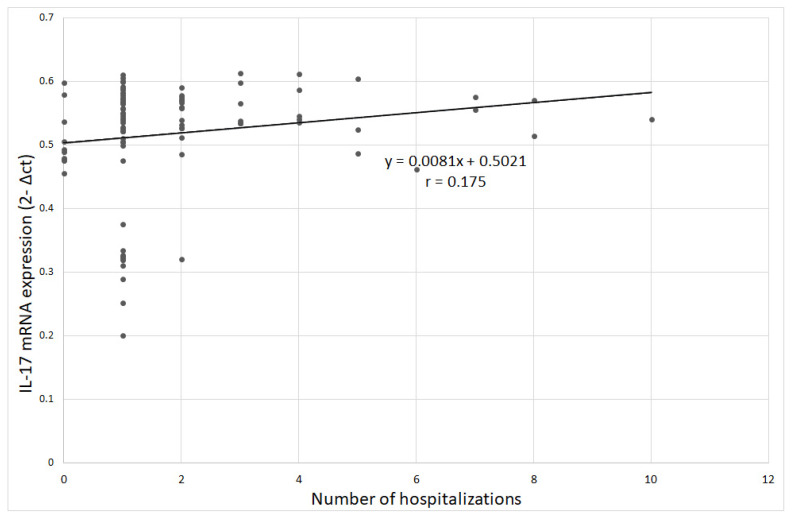
Correlation between IL-17 gene mRNA expression and number of hospitalizations;. r-Pearson’s linear correlation coefficient.

The analysis of the relationship between IL-17 mRNA expression and clinical variables in the group of patients with depression did not show any statistical significance. There was a tendency towards a significant correlation between IL-17 mRNA expression and the number of hospitalizations (Spearman’s rank correlation coefficient ρ = 0.199; *p* = 0.053), indicating that higher IL-17 mRNA expression was observed in patients with more hospitalizations (Figure 9). Other relationships were not statistically significant. The correlation between IL-17 gene mRNA expression and age was ρ = 0.163, *p* = 0.114.

## 4. Discussion

The latest comprehensive immune-inflammatory theory of major depression considers that increased IL-17 and Th17 activation participates in immune responses and neuro-immune toxicity [37]. There is evidence that depression is accompanied by increased expression of IL-17 [38,39]. IL-17, a pro-inflammatory cytokine, is a member of the cytokine family comprising IL-17A, IL-17B, IL-17C, IL-17D, IL-17E (IL-25) and IL-17F [22,23,24] and it was found before, to be elevated in the serum of patients suffering from depression. Tsuboi et al., 2018 identified increased serum levels of IL-17A protein among high depressive women in comparison with low depressive female participants [40]. Chen et al., 2011 and Davami et al., 2016 in turn, showed elevated protein levels of IL-17 in the serum of depressed patients (male/female) compared to healthy controls [41,42]. While the studies cited above show that depression is accompanied by an increase in the IL-17 protein in the serum, our results have shown that the expression of the IL-17 gene is also increased in the serum of depressed patients. These findings further confirm that this pro-inflammatory cytokine may be a biomarker of depression.

Obesity, like depression, is now a serious public health problem. Moreover, there is increasing evidence that depression and obesity are comorbid disorders [13,43,44,45]. This correlation seems to be bidirectional: obesity increase the risk of developing depression and depression increases the risk of developing obesity [43]. The biological link between obesity and depression has not been defined in full, although obesity may contribute by activating inflammatory pathways [13,46,47]. Furthermore, clinical studies showed the pathogenic role of IL-17 producing cells in the mechanisms underlying inflammation in the obesity and the obesity-related inflammatory diseases [20]. A study performed by Sumarac-Dumanovic et al., 2009 showed that obese women exhibited increased levels of circulating IL-23/IL-17 [47].

In our study, BMI does not affect IL-17 expression in patients with depression. However, the highest average of IL-17 protein level was observed in obese patients and in obese control subjects. Our findings are in line with the results of Bugge et al., 2018, who did not observe any significant correlation between IL-17 protein serum and BMI [48]. To our knowledge, our study is the first to analyze the correlation between IL-17 levels, obesity and depression, and it indicates that IL-17 or Th17 activation are unlikely to be a shared pathway between depression and obesity. Further studies on a larger group of patients should be performed to confirm these results.

Interestingly, a significant correlation was found between the serum level of IL-17 protein and the number of hospitalizations of patients. Further analysis of data did not show a significant correlation between increased IL-17 protein levels and the number of depressive episodes or duration and severity of the disease, which suggests that other factors than depression may underlie the impact of hospitalization. One of the largest population-based prospective studies indicated an increased risk for mood disorders after hospitalization because of autoimmune diseases and infections [49]. This suggests that a detailed analysis of the medical condition of patients should be performed to fully characterize the biological link between pro-inflammatory cytokine expression, depression and obesity.

Interleukin 17 is also involved in other autoimmune-related diseases [29,39]. Modern immunotherapies affecting this cytokine are used in the management of psoriasis and rheumatoid arthritis (RA) [50,51]. Some studies reveal that biological agents targeting IL-17, used in patients with autoimmune diseases, may have a clinical efficacy in the treatment of depressive disorders [29,52,53,54,55]. This may be related to the general improvement of the clinical condition and reduction of symptoms in this group of patients, but also to the possible common inflammatory background of depression and autoimmune diseases. Further research should be conducted to evaluate the effects of IL-17A inhibition in depressed patients, taking into account other common comorbidities, such as metabolic syndrome and obesity.

## 5. Conclusions

To conclude, our studies indicate that depression is accompanied by increased IL-17 mRNA and protein level in serum, thereby confirming the important role of IL-17 production in depression and its possible use as a biomarker of depression. Moreover, our research has shown no correlation between BMI and serum IL-17 levels in depressive patients, which may indicate that IL-17 is not a shared pathway between obesity and depression. This suggests that a detailed analysis of the medical condition of patients should be performed to fully characterize the biological link between pro-inflammatory cytokine expression, depression and obesity.

## Figures and Tables

**Table 1:** The demographic and baseline characteristics.

Variable		Group										Means Comparison
		Study					Control					
**Gender**		***n***		**%**			***n***		**%**			χ2 = 0.298; *p* = 0.585
	**M**	27		28.4			7		23.3			
	**F**	68		71.6			23		76.7			
		**min**	**max**	**$\bar{x}$**	**SD**	**V(%)**	**min**	**max**	**$\bar{x}$**	**SD**	**V(%)**	**Z = 4.208;** ***p*** = 0.000 *
**AGE [yrs]**		18	63	47.2	10.5	22.3	23	53	37.7	10.5	27.7	
**BMI [kg/m^2^]**		16	48	25.1	4.9	19.5	19.3	33.9	24.5	3.9	15.9	Z = 0.402; *p* = 0.688
**Duration of depression [yrs]**		1	30	5.5	5.8	105.8						
**No. Depressive episodes**		1	20	4.2	4.4	105.6						
**No. hospitalizations**		0	10	1.9	1.9	100.1						
**HDRS**		7	37	21.9	6.8	30.8						

M: male; F: female; BMI: Body Mass Index; HDRS: Hamilton Depression Rating Scale; *n*: number; %: percentage; min: minimum value; max-maximum value; $\bar{x}$-arithmetic
average; SD-standard deviation; V (%)-coefficient of variation; χ2-chi^2^
test result; Z-Z score (for Mann–Whitney test); *p*-statistical
significance; *-statistically significant.

**Table 2:** Interleukin 17 expression in studied groups.

Variable	Group										Means Comparison
	STUDY					CONTROL					
	min	max	$\bar{x}$	SD	V(%)	min	max	$\bar{x}$	SD	V(%)	
**IL-17 mRNA (2-Δct)**	0.201	0.613	0.518	0.089	17.2	0.048	0.393	0.238	0.070	29.5	Z = 7.934; *p* = 0.000 *
**IL-17 (ng/mL)**	123	449	309.1	79.5	25.7	44	143	104.2	25.1	24.1	Z = 8.151; *p* = 0.000 *

min: minimum value; max: maximum value; $\bar{x}$: arithmetic average; SD: standard deviation; V (%): coefficient of variation; Z: Z score (for Mann–Whitney test); *p*: statistical significance; *: statistically significant.
